# Effects of Aging on Determinants of Endurance Performance in Women Masters Athletes: A Scoping Review

**DOI:** 10.3390/healthcare14081080

**Published:** 2026-04-17

**Authors:** Danica Vangsgaard, Misa Noumi, K. Alix Hayden, Patricia K. Doyle-Baker

**Affiliations:** 1 Human Performance Laboratory, Faculty of Kinesiology, University of Calgary, Calgary, AB T2N 1N4, Canada; danica.vangsgaard@ucalgary.ca (D.V.); misa.noumi@ucalgary.ca (M.N.); 2 Libraries and Cultural Resources, University of Calgary, Calgary, AB T2N 1N4, Canada; ahayden@ucalgary.ca; 3 Alberta Children’s Hospital Research Institute, University of Calgary, Calgary, AB T2N 1N4, Canada; 4 O’Brien Institute of Public Health, University of Calgary, Calgary, AB T2N 1N4, Canada

**Keywords:** age-related decline, women, masters athlete, menopause, endurance sport, sport performance, aerobic capacity, lactate, economy

## Abstract

**Background/Objectives:** Masters athletes are adults aged ≥40 who compete in sport, exhibiting superior physical function and healthier aging than their sedentary peers. However, even highly trained masters athletes experience age-related performance declines. Women masters athletes represent a growing yet understudied population who may face unique physiological challenges. This scoping review synthesizes literature from 1984 to 2024, examining the impact of age and menopause on determinants of endurance performance in women masters athletes. **Methods:** Following JBI scoping review methodology, six databases were searched (Medline, Embase, Central, CINAHL, SPORTdiscus, Scopus). Studies were evaluated for population characteristics, methodological approaches, and physiological determinants of performance (i.e., aerobic capacity, lactate kinetics, and exercise economy). **Results:** Twenty-nine studies were included. Most (*n* = 28) assessed aerobic capacity, reporting declines between 0.36 and 0.84 mL·kg^−1^·min^−1^·year^−1^ (0.5–2.4%·year^−1^). These reductions were primarily associated with decreased cardiac output followed by changes in body composition. Training volume emerged as a predictor of aerobic capacity, but the effects of menopause were unclear. Findings on lactate kinetics and exercise economy were mixed but preliminary research indicated that lactate threshold relative to VO_2_max generally increased, peak lactate remained stable and energy cost increased with age. Fitness and health characteristics among women athletes differed from sedentary populations, emphasizing the need for athlete-specific data to support training and health decisions. **Conclusions:** Aging is associated with decreased aerobic capacity and variable changes in lactate kinetics and exercise economy. While training volume may attenuate performance decrements, the impact of menopause remains uncertain, underscoring the need for longitudinal research to better support this growing segment of the population.

## 1. Introduction

Participation in sport among women and girls is at an all-time high [1], including among those above the age of 40—namely, masters athletes [2,3]. Engaging in sport provides significant physical and mental health benefits, supporting overall wellbeing and quality of life [4,5,6]. As a result, masters athletes generally maintain better function than their age-matched sedentary peers and are often seen as models of healthy aging [7,8,9].

This growing participation has been accompanied by increased sport science research on masters athletes, especially in endurance sports such as triathlon, swimming, cycling, and running [3,10,11,12,13]. The popularity of these events provides a large data pool for studies examining the unique physiological and performance characteristics of masters athletes [3]. Yet, despite continued engagement in endurance sport, aging is associated with decreased performance, even in the most capable athletes [8,14].

Several studies have quantified age-related declines in running [11,12,15], swimming [11], and triathlon [9,16]. World records and cross-sectional comparisons show modest decreases starting around age 35 with accelerated declines beginning around ages 60–70 [11,14,16,17]. These changes are observed in both men and women and have been linked to a variety of physiological and training factors [11,14].

Endurance performance is commonly quantified through three central determinants: aerobic capacity (i.e., maximal, VO_2_max, or peak oxygen consumption, VO_2_peak), lactate kinetics, and exercise economy (i.e., oxygen cost or energy cost at submaximal exercise intensity) [18]. Although age-related declines in endurance performance are commonly attributed to decreased aerobic capacity [11], the reality is more complex and a holistic approach is needed to fully understand the underlying mechanisms.

While many studies have explored age-related declines in endurance performance, comparatively few reviews [3,19] or meta-analyses [20] have summarized these effects in women masters athletes. Meanwhile, existing summaries are often limited by small sample sizes, cross-sectional designs, non-athlete populations or a narrow focus on a single determinant of endurance performance [21].

The inadequate research on age-related decline in women athletes reflects the broader issue of “invisible sportswomen” in sport science [2,22]. Moreover, many studies including women rely on data from mixed cohorts [2,15], preventing careful analyses of women-specific experiences. Namely, the impact of menopause on endurance performance remains unclear [2] despite its serious and well-documented short- (e.g., hot flashes, mood changes, etc.) and long-term health effects (e.g., bone density loss, cardiovascular risk, cognitive changes, urogenital symptoms, etc.) [23,24]. Given that nearly all women experience menopause and many face related health complications, this knowledge gap poses significant health and performance concerns. Therefore, the primary purpose of this scoping review was to explore what is known about the impact of age on the determinants of endurance performance in women masters athletes. The secondary purpose was to explore what is known about the role of menopause in mediating these age-related changes.

## 2. Materials and Methods

The Population/Concept/Context (PCC) framework [25] guided the inclusion and exclusion criteria (Table 1). Articles were included if participants were women or female (aged 40 to 65) who were endurance athletes (runners, cyclists, swimmers and/or triathletes) classified as Tier 2 or above as per the Participant Classification Framework [26]. This classification requires athletes to train ≥ 3 per week, compete locally or above, and train with intent to compete [26]. These sports were selected based on preliminary searches indicating sufficient literature within these disciplines. Studies including other endurance sports (e.g., rowing) did not meet the remaining inclusion criteria. In addition, studies had to include at least one key determinant of endurance performance (i.e., aerobic capacity, lactate kinetics, exercise economy) with changes reported over time, either as a longitudinal or as a cross-sectional study comparing younger to older athletes. Articles with men athletes or sedentary women were included if the data could be stratified to allow analysis of eligible women masters athletes. Cross-sectional studies comparing younger to older athletes were considered if at least one age group fell within the 40 to 65 age criteria. This age range was chosen to align with common definitions of masters athletes in sport science research [3], while focusing our search on the years surrounding menopause, thus supporting our secondary objective.

Given the relative lack of sport science research on women masters athletes, a scoping review was chosen to broadly map and characterize the state of the literature [25,28]. This style of review was chosen to identify key concepts and knowledge gaps, and inform future, more focused investigations. The JBI (formerly Joanna Briggs Institute) methodology for scoping reviews [25] was followed and we reported the process according to the Preferred Reporting Items for Systematic Reviews and Meta-Analysis extension for Scoping Reviews (PRISMA-ScR) (Appendix A) [29]. The three-step approach included a preliminary search of Google Scholar, Medline, and the Cochrane Database of Systematic Reviews and JBI Evidence Synthesis. Although several reviews examined age-, sex- and gender-related changes in endurance or strength outcomes, no reviews specifically focused on women masters athletes [20,30,31,32] or addressed menopause as a subtopic in this field. Nevertheless, an adequate number of seed articles [16,33,34,35,36] were identified to support the feasibility of conducting a scoping review. Next, text words from the titles and abstracts as well as subject headings from seed articles informed the development of a comprehensive Medline search strategy (Appendix B). The search included four concepts: women, age, masters athletes, and endurance performance. Each concept included keywords and subject headings. The Medline search was translated to other databases where keywords were the same across all databases, and subject headings were responsive to the controlled vocabulary of each database. The preliminary protocol was conducted in accordance with an a priori protocol [25] and registered on 18 December 2024, with the Open Science Framework Registries OSF (https://osf.io/dfjcv/overview; accessed 15 December 2025).

### 2.1. Search Strategy

The following databases were searched in September 2024: Medline All (Ovid), Embase (Ovid), Central (Wiley), CINAHL Plus with Full Text (Ebsco), SPORTdiscus with Full Text (Ebsco), and Scopus (Elsevier). Additionally, the reference lists of all included sources were screened to identify further studies. There were no restrictions on publication date or language. Our search was supported by a librarian (K.A.H.) and retrieved database records were uploaded into Covidence [37]. The full search strategy for each database can be found under Appendix B.

### 2.2. Study Selection

Following the search, all identified citations were collated and uploaded into Covidence (Covidence systematic review software, Veritas Health Innovation, Melbourne, Australia. Available at www.covidence.org.) [37]. After duplicates were removed, all reviewers (D.V., M.N., P.K.D.-B.) independently conducted a pilot test of 50 randomly selected records (titles and abstracts) against the inclusion criteria. Interrater agreement was above the suggested 75%. After the pilot test, D.V. and M.N. screened all retrieved titles and abstracts to assess their eligibility. Full texts of potentially relevant citations were then reviewed by D.V. and M.N. to determine final inclusion. Disagreements at any stage were resolved through discussion with P.K.D.-B. and reasons for exclusion at the full-text stage were documented. Following consultation with P.K.D.-B., one study involving “endurance-trained” women was included given the high fitness and training frequencies (i.e., ≥3 sessions per week [26]; Table 1) and the article’s inclusion of menopause-related metrics, despite not explicitly including women masters athletes [38].

### 2.3. Data Extraction

During the pilot screening phase, the data extraction form was tested with identified seed studies (Appendix C). It was modified and refined as necessary throughout the data extraction process. Extracted data included specific details about the participants, concept, context, study methods, and key findings relevant to the review questions. Through the data extraction process, additional columns were added to increase detail about exercise protocols and performance metrics beyond the three primary performance determinants. Articles screened prior to the addition of new data extraction criteria were revisited to capture the relevant information. Data extraction was conducted by D.V. except for one Japanese-language article [39] which was reviewed by M.N., who is fluent in Japanese. Data extraction was verified by P.K.D.-B. After extraction, D.V. and P.K.D.-B. discussed prevalent themes and established a logical flow for presenting findings. We were unable to classify participants as per the STRAW+10 framework (Stages of Reproductive Aging Workshop) outlined in our protocol [24] because few studies provided details on their method of identifying menopause status.

## 3. Results

**Figure 1:**
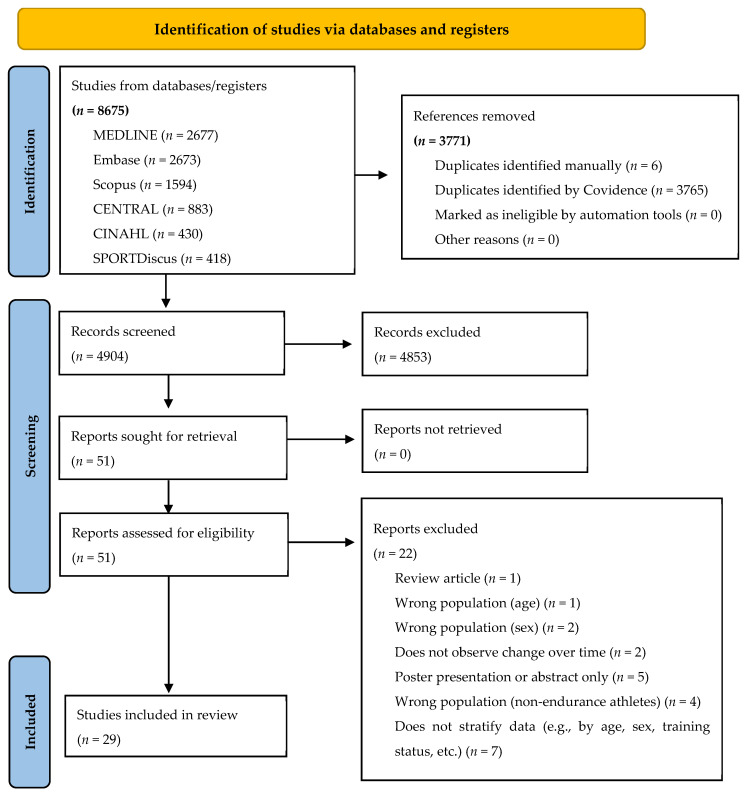
PRISMA 2020 flow diagram for new systematic reviews [29] (Appendix A).

The initial search identified 8675 records, of which 3771 duplicates were removed, leaving 4904 for title/abstract screening. Of these, 4853 were excluded, with 51 advancing to full-text review. Only 29 studies met inclusion criteria for analysis. The most common exclusion reason was lack of stratified data (Figure 1).

The earliest article was published in 1984 and the most recent in 2022 (Table 2). Most articles were published in North America (*n* = 21), 19 of which were published in the United States. Study designs were either cross-sectional (*n* = 25) or longitudinal (*n* = 4). Most studies included athletes between the ages of 40 and 70, but one study included athletes as young as 13 [40] and one as old as 90 [41]. Twelve studies discussed menopause [34,38,39,42,43,44,45,46,47,48,49,50], although the extent to which it was discussed varied greatly. Menopause was typically defined as ≥12 months of amenorrhea [38,42,44,46] but some required participants to be ≥24-month from last menses [45]. One study classified menopause status using the comprehensive Notelovitz framework [49,51].

Aerobic capacity was included in every article except one (*n* = 28) [52]. Seven articles presented measures of lactate [34,35,52,53,54,55,56] and three mentioned exercise economy [49,57,58]. Other articles reported metrics such as VO_2_ at estimated anaerobic threshold [59], glucose response to endurance exercise [54], and lipid profiles of endurance-trained women [39,44].

Age-related decline in aerobic capacity was presented in several ways, including regression equations, absolute changes (mL·kg^−1^·min^−1^·year^−1^), or percent changes (%·year^−1^) (Table 3). Lactate parameters were most frequently presented as peak lactate [34,35,52,54,55], lactate threshold [35,55,56] or lactate threshold as a percentage of VO_2_max [35,55,56,57]. Exercise economy was typically presented as oxygen cost [49,57,58] or energy cost [58] of submaximal exercise.

Aerobic capacity was assessed predominantly using treadmill [34,35,38,39,42,43,44,45,46,47,48,54,55,56,57,58,60,61,62,63,64,65] and cycle ergometer-based indirect calorimetry [41,50,53,60,61]. Twenty-three studies determined aerobic capacity by measuring VO_2_max. Most of these studies (*n* = 20) included standardized criteria to confirm attainment of a true physiological maximum, such as a plateau in VO_2_ despite increasing work rate, respiratory exchange ratio (between 1.05 and 1.15, heart rate within 10% of age-predicted maximum, and a rating of perceived exertion near maximal effort [34,35,39,40,43,44,45,47,48,49,50,54,55,56,57,61,62,63,64,65]. Studies lacking standardized criteria for aerobic capacity reported VO_2_peak (*n* = 5), representing the highest oxygen consumption reached during an exercise bout [38,41,42,58,60]. While VO_2_max and VO_2_peak are distinct concepts [66], they are referred to collectively as aerobic capacity throughout this review.

Testing protocols were similar across studies. For example, several treadmill-based protocols used a modified Balke test that began at 2.5 miles per hour with incremental increases in grade by 2% and speed by 0.5 miles per hour every two minutes until volitional exhaustion [34,35,43,57]. Lactate concentrations were assessed either by intravenous sampling or portable devices [34,52,53,54]. Lactate-related metrics varied across studies, with some authors reporting peak exercising concentrations and others assessing lactate threshold as a percentage of VO_2_max, among other metrics. Few studies collected lactate data outside of treadmill-based assessments [52].

Exercise economy, referring to the oxygen or energy cost required to perform submaximal exercise, was mentioned in three studies [49,57,58], with in-depth analysis limited to just one study [58].

Body composition was measured using hydrostatic weighing [34,43,45,47,48,54,55,65], skinfold calipers [42,46,48,62,64] and dual-energy X-ray absorptiometry [38,43,48,56,62,63]. Body composition assessments were used to quantify bone mineral density, fat mass, and fat-free mass, and to facilitate discussions on the relationship between body composition and aerobic capacity [43,47,54,55].

**Table 3:** Studies by author, determinant of endurance performance, primary study objective, and conclusion.

Author (Year)	*n*	Age Range	Determinant			Primary Objective	Conclusion
			AC	LK	EE		
Rainville (1984) [46]	20	41–61	X	–	–	Impact of training and menopause on cholesterol levels.	Cholesterol profiles became less favourable after menopause, but this was attenuated by training.
Vaccaro (1984) [65]	42	20–69	X	–	–	Body composition and physiological response to exercise in swimmers.	Highly trained swimmers had more favourable body composition, but VO_2_max declined regardless of training status.
Goldfarb (1986) [54]	35	25–72	X	X	–	Age effects on glucose and blood lactate in trained women.	Glucose response to exercise changed above age 60, but peak lactate did not.
Higuchi (1990) [44]	33	35–57	X	–	–	Impact of age on lipid and lipoprotein profiles in runners.	Lipid profiles become less favourable after menopause, but this was attenuated by training.
Itoh (1992) [39]	33	~43–53	X	–	–	Compare dietary profiles of trained and sedentary females.	Trained women reported better dietary profiles.
Wells (1992) [49]	49	35–70	X	–	X	Impact of age, training, and menopause on cardiorespiratory fitness.	Menopause did not account for decline in VO_2_max after adjusting for age and training status. Training attenuated decline in maximum HR but not VO_2_max.
Koda (1994) [40]	60	13–68	X	–	–	Impact of age on VO_2_max in Japanese swimmers.	VO_2_max declined with age in trained individuals but remained higher than in untrained individuals.
Ryan (1996) [63]	43	18–69	X	–	–	Impact of training on adverse age-related changes in body composition.	Training may preserve fat free mass and reduce intraabdominal adipose tissue with age.
Hunt (1997) [45]	39	21–72 †	X	–	–	Impact of age, training status, and hormone status on blood pressure.	Blood pressure increased by similar amounts in trained and sedentary women, but via different mechanisms.
Proctor (1997) [62]	16	19–72	X	–	–	Change in VO_2_ per kg of limb muscle mass.	VO_2_ per kg of muscle mass decreased with age due to decreased O_2_ delivery.
Tanaka (1997) [64]	84	20–75 †	X	–	–	Rate of VO_2_max decline between trained and sedentary women.	Absolute rate of VO_2_max decline was greater in trained compared to sedentary women, but relative rate VO_2_max was similar between groups.
Van Pelt (1997) [47]	38	18–73 †	X	–	–	Decline in resting metabolic rate between trained and sedentary women.	Resting metabolic rate declined in sedentary but not trained women.
Proctor (1998) [61]	14	24–72	X	–	–	Impact of age and sex on the cardiac output–VO_2_ relationship.	Cardiac output–VO_2_ slope was similar between ages and sexes.
Van Pelt (1998) [48]	59	18–73 †	X	–	–	Impact of training on age-related increases in body mass and adiposity.	Body mass did not increase and the increase in adiposity was attenuated in the trained women.
Wiebe (1999) [50]	23	20–61	X	–	–	Physiological reasons for age-related VO_2_max decline in trained women.	VO_2_max decline was driven by decreased cardiac output (decreased SV and HR), but arteriovenous difference was preserved.
Wiswell (2000) [55]	57	40–60+ †	X	X	-	Impact of age on LT and its relationship with performance.	LT as a % of VO_2_max increased with age but VO_2_max was a better predictor of performance.
Hawkins (2001) * [34]	49	40–60+ †	X	X	–	Age, sex, and training effects on VO_2_max.	VO_2_max decline was attenuated by MHT and maintained by training volume in women.
Wiswell (2001) [56]	82	40–77	X	X	–	Describe training load, fitness, strength, and body composition in masters athletes.	Decline was apparent in most examined metrics, but rates of decline did not differ between sexes.
Eskurza (2002) * [57]	16	40–78 †	X	–	X	Impact of training status on rate of VO_2_max decline.	Absolute rate of VO_2_max decline was similar between sedentary and trained women when training was maintained.
Hawkins (2003) * [43]	41	~46–65	X	–	–	Effects of chronic exercise and MHT on BMD.	No significant bone loss in runners, regardless of MHT use, provided sufficient dietary Ca^2+^.
Marcell (2003) * [35]	23	39–75 †	X	X	–	Age-related change in LT and its impact on performance.	LT as a % of VO_2_max increased with age, but LT was not predictive of performance.
Benelli (2007) [52]	56	40–79	–	X	–	Measured peak, post-competition lactate in masters swimmers.	Females reported lower peak lactates compared males and did not show age-related declines in peak lactate.
Brown (2007) [53]	20	16–54	X	X	–	Age-related VO_2_max and power output decline in cyclists.	VO_2_max and power at VO_2_max declined in males and females, but underlying mechanisms may be sex-specific.
Burtscher (2008) [59]	3	35–70+	X	–	–	Relationship between VO_2_ at anaerobic threshold and performance.	VO_2_ at anaerobic threshold declined similarly in males and females but can be maintained until 45–49 years.
Copeland (2014) [42]	12	30–62	X	–	–	Endocrine response to an ultramarathon in premenopausal and postmenopausal runners.	Both groups experienced a significant increase in estradiol, but there was a greater relative increase in the postmenopausal group.
Bagley (2019) [41]	11	37–90 †	X	–	–	Age-related performance changes in power versus endurance athletes.	Peak anaerobic and aerobic power declined 7–14% per decade in all groups.
Lee (2019) [58]	13	40–71 †	X	–	X	VO_2_peak and running economy (i.e., EE) in masters runners.	VO_2_max declined with age. Energy cost of running increased in females but not males.
Rael (2021) [38]	98	~26–51	X	–	–	Impact of different hormonal milieux on cardiorespiratory fitness.	Age and estrogen loss was linked to VO_2_max decline post menopause, but this can be attenuated by training.
Petek (2022) [67]	83	18–60	X	–	–	Generated cardiopulmonary exercise test reference ranges for endurance athletes.	Developed VO_2_peak prediction equations for endurance athletes.

Note: AC, aerobic capacity (i.e., VO_2_max or VO_2_peak); EE, exercise economy; LK, lactate kinetics; SV, stroke volume; HR, heart rate; LT, lactate threshold; MHT, menopause hormone therapy; BMD, bone mineral density. *N* = total number of well-trained athletes (including younger age groups and premenopausal women). Age range generally refers to women athlete populations only, except ~ indicates mean ages for women athletes (article did not provide age ranges) and † indicates age range of entire population (article did not specify range for women athletes). * Longitudinal study.

## 4. Discussion

This scoping review examined current knowledge on how aging and menopause affect determinants of endurance performance in women masters athletes. Our discussion focuses on the physiological basis of three primary determinants [11,18] and explores key factors such as cardiac output, body composition, menopause, and training volume.

### 4.1. Aerobic Capacity

Age-related decline in aerobic capacity was discussed in 28 studies [34,35,38,39,40,41,42,43,44,45,46,47,48,49,50,53,54,55,57,58,59,60,62,63,64,65,68]. At the group level, aerobic capacity in women athletes declined by 0.36 to 0.84 mL·kg^−1^·min^−1^·year^−1^. Some authors also reported declines by subgroup (e.g., age group [34] or training volume [57]). In relative terms, aerobic capacity declined by 0.5–2.4% per year with younger athletes showing slower rates and older athletes showing faster rates of decline, demonstrating the curvilinear change discussed above (Table 4) [8,11,14,17]. Longitudinal studies, which capture within-person changes, reported higher rates of decline compared to cross-sectional designs [34,35,43,57]. This discrepancy may reflect methodological limitations inherent to cross-sectional studies, including selection bias (e.g., fitter individuals more likely to participate) as well as genetic and other group differences [47,48,50]. Nonetheless, each study design has its benefits: cross-sectional designs showcase the athletic potential of women masters athletes while longitudinal designs better reflect typical aging processes. However, explanations for differences between cross-sectional and longitudinal estimates remain partly speculative, and further work is needed—particularly in women masters athletes—to clarify the underlying mechanisms [65].

Women masters athletes exhibited higher absolute aerobic capacities than their sedentary peers, but they also tended to experience higher absolute rates (mL·kg^−1^·min^−1^·year^−1^) of decline [64], likely due to greater baseline fitness and greater reductions in training volume [32]. However, when expressed as a percent loss per year, the relative rate (%·year^−1^) of decline was comparable between athletes and sedentary women [34,57,64]. To illustrate this, Eskurza et al. [57] reported declines of 0.84 mL·kg^−1^·min^−1^·year^−1^ in trained women, compared to 0.4 mL·kg^−1^·min^−1^·year^−1^ in sedentary women. While this difference may seem jarring when expressed in absolute terms, this translates to similar relative declines of 1.8%·year^−1^ and 1.5%·year^−1^ in trained and sedentary women, respectively [54]. This age-related decline appears to be primarily associated with decreased cardiac output followed by changes in body composition [34,41,64]. Training volume emerged as a key and modifiable determinant of aerobic capacity where women who maintained training volume attenuated age-related declines [34,57]. Given the impact of estrogens on the cardiovascular system, hormonal changes may also be influential, but these effects—and menopause more broadly—were rarely studied [42].

#### 4.1.1. Cardiac Output

Aerobic capacity is determined by cardiac output (i.e., volume of blood ejected by the heart per minute) and the arteriovenous oxygen difference (∆a-vO_2_) (i.e., capacity of the tissue to extract oxygen from the arterial blood) [69]. Cardiac output is the product of heart rate and stroke volume, both of which may be influenced by age-related changes [34,49,57]. Maximal heart rate consistently decreased with age regardless of fitness status [34,57,60,61,64], likely due to changes in intrinsic cardiac pacemaker function [70]. This decline is well established and considered a significant physiological factor underlying age-related reductions in aerobic capacity.

The association between stroke volume and aging was more nuanced. While stroke volume decreased with age in sedentary women [45], findings among women masters athletes were less consistent. Some studies reported no differences between younger and older athletes [45], except at high exercise intensities (i.e., 70% of VO_2_peak) [61]. Conversely, Wiebe et al. [50] reported lower stroke volumes in older (aged 40–63) versus younger athletes (aged 20–29), attributing these differences to alterations in cardiac dimensions such as increased end-systolic volume and/or decreased end-diastolic volume. Overall, findings were mixed with some studies suggesting that stroke volume may decrease with age, while others showed that endurance training may attenuate these effects [7,71].

#### 4.1.2. Arteriovenous O_2_ Difference (∆a-vO_2_)

The contribution of ∆a-vO_2_ to age-related reduction in aerobic capacity appeared to be limited [50,71]. Although decline in ∆a-vO_2_ was observed in sedentary adults, trained individuals generally preserved their ability to extract oxygen at the muscular level [62,71]. Proctor and Joyner [62] demonstrated that decreases in aerobic capacity normalized to muscle mass were predominately attributed to diminished cardiac output—and consequently reduced oxygen delivery—rather than to impaired ∆a-vO_2_. Compared with ∆a-vO_2_, these findings suggest that reduced cardiac output is more strongly associated with age-related decline in aerobic capacity among women masters athletes.

#### 4.1.3. Body Composition

Body composition was identified as another factor contributing to changes in aerobic capacity. Wiswell et al. [56] reported that lean body mass positively and body fat percentage negatively correlated with aerobic capacity in women runners. These associations likely reflect the role of lean body mass in supporting cardiac output as well as the increased energetic cost of movement related to greater fat mass [72]. Although age-associated decreases in fat-free mass and increases in fat mass [34,35,39,44,45,46,47,48,56,57,61,62,64] might be expected to significantly reduce aerobic capacity [48,64], their effect was generally modest: normalizing aerobic capacity to fat-free mass attenuated but did not fully account for the decline. Therefore, while body composition may be a contributing factor, it is likely not the most important contributor to age-related decline in aerobic capacity [62,64]. These findings suggest that focusing on body composition may be less effective for maintaining fitness than other interventions such as training among women masters athletes [73].

#### 4.1.4. Menopause and Sex Hormones

Menopause and sex hormones represent an underexplored area of health and performance in women masters athletes [42]. Given the cardioprotective effect of estrogens [23], menopause-related declines in estrogen may by hypothesized to adversely affect cardiovascular functions such as aerobic capacity [34,38]. Despite this, few studies quantified estrogen and none considered progesterone levels or the complex interactions between multiple hormones [2]. Research on menopause hormone therapy (MHT) could elucidate the influence of sex hormones on endurance performance, but few studies controlled for or explicitly examined MHT usage [34]. For example, Wells et al. [49] did not account for MHT despite 14 of their 18 postmenopausal participants using it. While no independent effect of menopause was observed in their study, MHT may have masked such an effect [49]. Moreover, no studies considered the impact of menopause symptoms on exercise performance, despite their known negative effects [74]. This highlights a notable lack of literature exploring the lived experiences of women masters athletes during menopause. These gaps leave women masters athletes without clear evidence to guide decisions on hormonal health or menopause management. Future research should consider a more holistic approach by assessing hormonal status alongside menopause symptoms and their effects on quality of life, training, and performance.

Furthermore, generalizing findings from broader populations may be limited by physiological differences between women masters athletes and their sedentary peers. Specifically, sedentary women typically present with higher estrogen levels [44], potentially due to higher adiposity [73]. Similar differences also exist for aerobic capacity, bone mineral density, and muscle mass, whereby athletes tend to report higher values than their sedentary peers. Accordingly, declines in these parameters may be underrecognized in women masters athletes. Therefore, developing athlete-specific benchmarks is essential for identifying and managing health concerns in this population [60].

#### 4.1.5. Metabolism

Metabolic changes may contribute to declines in aerobic capacity. Menopause-related estrogen loss is associated with increased carbohydrate oxidation and reduced fat oxidation, potentially altering substrate utilization during exercise and promoting increased fat mass [63]. Nevertheless, endurance training appears to counteract many of these metabolic shifts, enabling women masters athletes to maintain high resting metabolic rates and show favourable lipid and glucose profiles compared to their sedentary peers [44,46,47,63]. Additionally, athletes demonstrate higher energy expenditure [45], supporting sufficient nutrient intake without adverse changes in body composition [39]. Although not directly examined, these healthier metabolic profiles likely facilitate aerobic fitness both by enabling sustained training volumes and enhancing the physiological systems integral to aerobic capacity.

#### 4.1.6. Training Volume

Lastly, training volume was identified as one of the most significant predictors of aerobic capacity [34,57,59]. Endurance training enhances cardiovascular fitness through central and peripheral adaptations, including ventricular hypertrophy, increased blood volume, and greater mitochondrial density [69,75]. Although women masters athletes exhibited higher aerobic capacities than sedentary women, they often experienced accelerated absolute declines due to higher baseline fitness and more substantial reductions in training volume [32,34,57]. Maintaining training status may thus mitigate age-related decline [76,77].

Current training research focuses predominantly on training volume. For example, Eskurza et al. [57] observed that endurance-trained women who reduced training volume experienced double the rate of absolute decline in aerobic capacity compared to endurance-trained women who maintained it (1.04 mL·kg^−1^·min^−1^·year^−1^ vs. 0.52 mL·kg^−1^·min^−1^·year^−1^). However, other training parameters, such as training intensity, warrant investigation as reductions in intensity may reduce both aerobic [34] and anaerobic capacity [78]. Furthermore, the training data examined here are largely observational, limiting causal inference. Comprehensive intervention studies are therefore needed to determine how volume, intensity and other training parameters interact to optimize endurance performance in women masters athletes [11,76]. Nevertheless, these findings underscore training as a practical, modifiable strategy for preserving aerobic capacity and promoting lifelong fitness.

### 4.2. Lactate Kinetics

**Figure 2:**
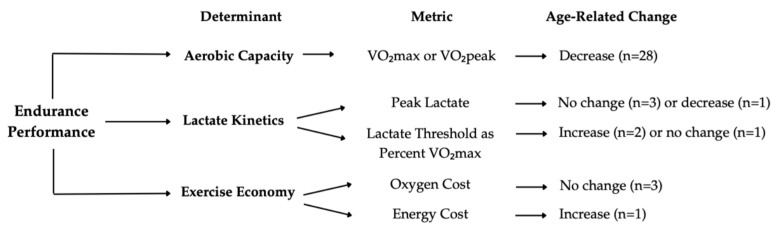
Summary of age-related changes in each determinant of endurance performance.

Compared to aerobic capacity, blood lactate parameters were less extensively studied. Most evidence indicated stable peak lactate concentrations with age in women masters athletes [34,52,54,64], whereas one study observed a decrease [31] (Figure 2). Notably, two conflicting studies were conducted by the same research group, using similar protocols and likely drawing from the same cohort of athletes [34,35]. Although VO_2_max and weekly running volume were comparable between datasets, unreported differences in training programs (e.g., intensity) may explain this discrepancy. Regardless, these findings highlight variability in age-related changes in peak lactate.

Meanwhile, although one study reported no change [52], two studies found that lactate threshold relative to VO_2_max increased with age (~0.3%·year^−1^) [35,55]. Though a rightward shift in the lactate curve has been historically thought to indicate improved fitness in young athletes, this shift seems to occur independently of training adaptations in masters athletes [35], suggesting that the relationship between lactate threshold and endurance performance may weaken with age [79]. Such changes in lactate kinetics have been proposed to reflect reductions in skeletal muscle mass, particularly loss of Type II fibers [35,52,53,78], although reductions in high intensity training may also contribute [78]. These findings highlight the need for longitudinal research on lactate kinetics and emphasize the importance of individualized, periodic lactate assessments when prescribing training zones in women masters athletes.

### 4.3. Exercise Economy

Research on exercise economy in women masters athletes was limited. Early investigations [49,57] reported no age-related decline in exercise economy; however, these studies reported oxygen cost, capturing only the aerobic demands of exercise. More recent work [80] has emphasized the importance of total energy cost which integrates contributions from all energy systems and thus offers a more comprehensive assessment of exercise economy. Using this broader approach, Lee et al. [58] observed that energy cost increased with age, potentially due to alterations in tendon stiffness, muscle fiber-type composition, and biomechanical factors such as stride mechanics [58,81]. Although interest in exercise economy is growing, the available evidence remains preliminary, highlighting the need to re-examine earlier findings using updated methods (i.e., total energy cost) and to investigate the factors influencing economy in women masters athletes.

### 4.4. Limitations

This scoping review identified several limitations in the existing literature. First, many studies were published over 25 years ago and relied on outdated methodologies, such as hydrostatic weighing for body composition assessment [34,43,47,48,54,55,65] and Douglas bags for aerobic capacity assessment [39,40]. Although these methods remain valid, differences in measurement techniques may limit comparability with more recent findings. In addition, menopause status and athlete competitive level were often poorly defined with few studies specifying standardized criteria for menopause [24] or athlete classification [26]. The absence of classification criteria further challenges cross-study comparisons and may contribute to heterogeneity in findings. Furthermore, most studies used cross-sectional designs, which are vulnerable to confounding and selection biases, limiting causal inference and generalizability to the broader population of women masters athletes. This issue is exacerbated by the small sample sizes present in many studies, particularly among older age groups. Finally, inconsistent and ambiguous terminology impeded nuanced discussion. For example, some authors used the blanket-term “estrogens” without specifying the type of estrogen, and gender-related terms were often used where sex-related terms may have been more appropriate.

### 4.5. Directions for Future Research

This review identified several critical gaps in the literature. While age-related decline in aerobic capacity is well characterized, considerably less is known about lactate kinetics and exercise economy. Future studies should prioritize these determinants while examining training parameters beyond volume (e.g., intensity) and clarifying the effects of menopause status and sex hormones on all three determinants of endurance performance. Lastly, athlete-specific normative data is needed not only to improve the identification of potential pathologies in women masters athletes, but also to enhance our understanding of optimal health and physiological aging in this population. Longitudinal and interventional designs that employ contemporary methodologies along with standardized classification criteria for menopause status and athlete competitive level will help address these gaps.

## 5. Conclusions

We reviewed 29 articles exploring the impact of aging on determinants of endurance performance in women masters athletes. These articles consistently reported an age-related decrease in aerobic capacity with women masters athletes maintaining substantially higher fitness than their sedentary peers, despite greater absolute and similar relative rates of decline. Although findings were mixed, peak lactate concentrations generally remained stable while lactate threshold as a percentage of VO_2_max increased with age. Preliminary research on exercise economy suggests that energy cost of running may increase with age, but further research is needed to confirm this finding.

Important gaps in the literature included menopause and hormone status, thus limiting our understanding of their contributions to endurance performance. Similarly, lactate kinetics and exercise economy were scarcely examined. Future longitudinal studies are needed to examine within-person changes in these key determinants of endurance performance. Finally, this review highlighted several physiological differences between women masters athletes and sedentary women, underscoring the need for athlete-specific normative data. As sport participation continues to rise among women masters athletes, further work is needed to examine the effects of menopause and evaluate strategies aimed at optimizing performance in this population.

Collectively, these findings highlight the complex interplay between aging, menopause, and the central determinants of endurance performance while providing direction for future research aimed at understanding and improving the performance of women masters athletes.

## Figures and Tables

**Table 1:** Inclusion and exclusion criteria by PCC category, sub-category, and criteria.

PCC Category	Sub-Category	Criteria
Population	Women *	Women or female participants, aged 40 to 65.
	Athletes	Endurance athletes (runners, cyclists, swimmers, triathletes).
	Competitive level	Classified as Tier 2 or above [26].
	Other	Men athletes or sedentary women included if stratified for women or female athletes aged 40–65.
Concept	Determinant of endurance performance	At least one key determinant of endurance performance (aerobic capacity, lactate kinetics, exercise economy) with change over time (longitudinal). Comparison of younger and older athletes (cross-sectional) included if at least one group aged 40–65.
	Menopause	Studies including relevant discussions on menopause.
Context	Design, Setting, Publication Type	No limits on study setting. Peer-reviewed literature only. Reviews, qualitative studies or opinion papers were excluded.
	Language, Year	No limits on publication date or language.

* Many included articles were published before clear distinctions between sex and gender were emphasized in sport science research, resulting in the use of gendered terms (e.g., women) where sex-specific language (e.g., female) may have been more appropriate [27]. While this review focuses on biological processes, the included studies rarely justified their use of “women” versus “female.” While we acknowledge that sex and gender are distinct and non-binary concepts, “women” is recognized as an inclusive term [22], including biological females as well as individuals who identify as women. As such, we use “women” throughout this review to align with and enable a cohesive discussion of the original sources.

**Table 2:** Study characteristics by location, year, design, determinant of endurance performance, and menopause status.

Study Characteristics	Category	Number (*n* = 29)
Location (Continent)	North America	21
	Europe	4
	Oceania	1
	Asia	3
Publication Year	1980–1984	2
	1985–1989	1
	1990–1994	4
	1995–1999	8
	2000–2004	6
	2005–2009	3
	2010–2014	1
	2015–2019	2
	2020–2024	2
Study Design	Cross-sectional	25
	Longitudinal	4
Endurance Determinant *	Aerobic Capacity	28
	Lactate Kinetics	7
	Exercise Economy	3
Menopause	Discussed	12
	Not discussed	17

Note: Lactate kinetics include various blood lactate metrics such as peak lactate, lactate threshold or % of VO_2_max. * Some studies included multiple endurance determinants (Table 3).

**Table 4:** Absolute and relative rates of decline in aerobic capacity in women masters athletes (reported as group means).

Author (Year)	Population	Age Range	Absolute Rate (mL·kg^−1^·min^−1^·year^−1^)	Relative Rate (%·year^−1^)
Vaccaro (1984) [65]	Swimmers	20–69	—	0.7
Wells (1992) [49]	Runners	35–70	0.58	—
Koda (1994) [40]	Swimmers	40–69	0.45	0.87
Tanaka (1997) [64]	Runners	20–75	0.57	0.97
Proctor (1998) [61]	Endurance athletes	24–72	—	~0.6
Hawkins (2001) [34]	Runners	40–73	—	0.5–2.4 *
Wiswell (2001) [56]	Runners	40–86	0.36	0.8
Eskurza (2002) [57]	Runners	51–63	0.84	1.8
Brown (2007) [53]	Cyclists	16–54	0.39	—

* 0.5% and 2.4% is the decline for the youngest and oldest age group, respectively.

**Figure A1:**
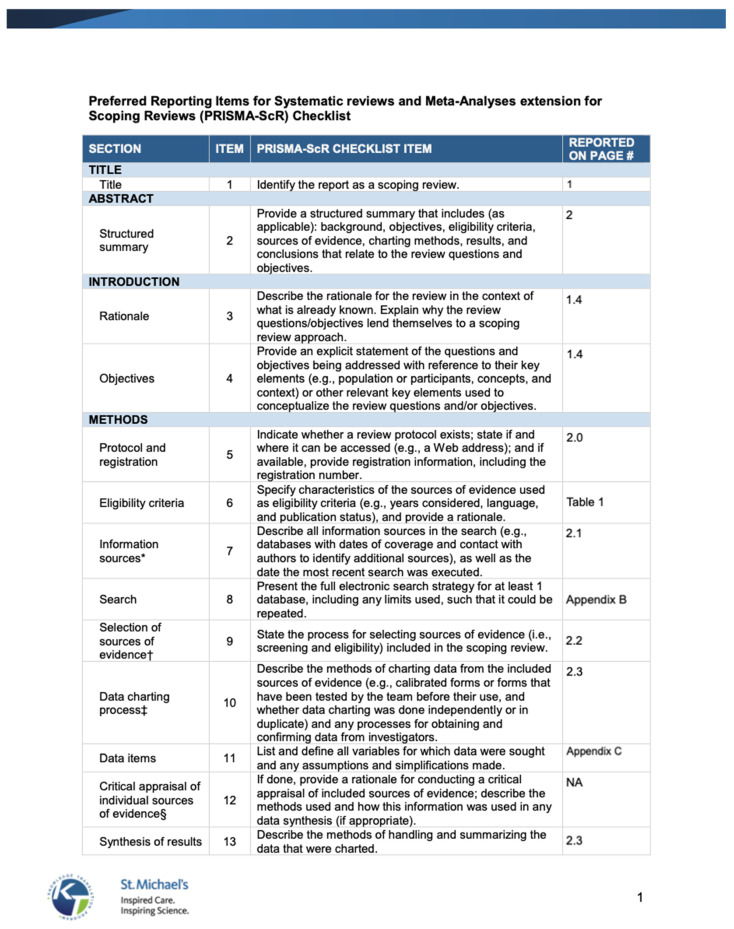

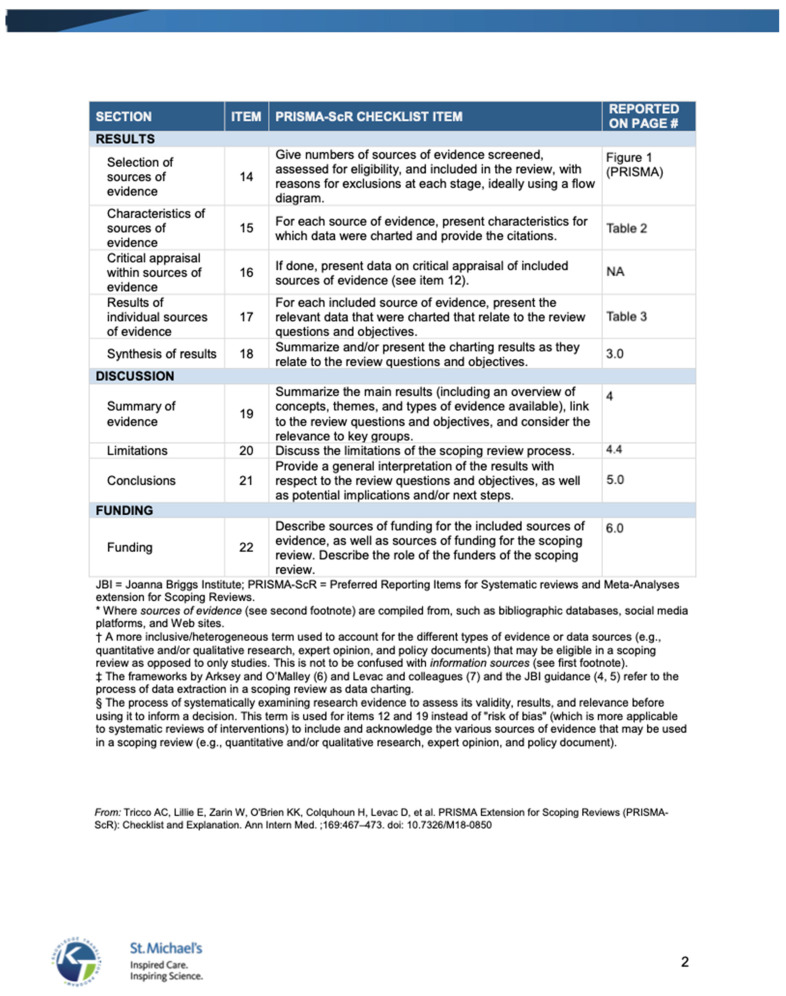
PRISMA-ScR Checklist [82].

## Data Availability

The data presented in this study are available on request from the corresponding author.

## Abbreviations

The following abbreviations are used in this manuscript:

AC	Aerobic capacity
BMD	Bone mineral density
EE	Exercise economy
HR	Heart rate
LK	Lactate kinetics
LT	Lactate threshold
MHT	Menopause hormone therapy
SV	Stroke volume
VO_2_max	Maximal oxygen consumption
VO_2_peak	Peak oxygen consumption
∆a-vO_2_	Arteriovenous difference

## Appendix B. Database Search Strategies

**Table A1:** Database: Ovid MEDLINE(R) ALL 1946 to 2024 September 13. Search Strategy.

#	Searches	Results
1	Female/	9,910,106
2	(female* or women* or woman*).tw,kf.	2,605,351
3	1 or 2	10,393,765
4	Age Factors/or Aging/or Middle Aged/	5,298,909
5	(aging or older).tw,kf.	819,576
6	menopause/or perimenopause/or postmenopause/	58,919
7	(“transitional menopause” or menopaus* or perimenopaus* or postmenopaus*).tw,kf.	109,752
8	((“35*” or “36*” or “37*” or “38*” or “39*”) adj2 (year* or age* or yr*)).tw,kf.	303,294
9	((“40*” or “41*” or “42*” or “43*” or “44*” or “45*” or “46*” or “47*” or “48*” or “49*”) adj2 (year* or age* or yr*)).tw,kf.	616,803
10	((“50*” or “51*” or “52*” or “53*” or “54*” or “55*” or “56*” or “57*” or “58*” or “59*”) adj2 (year* or age* or yr*)).tw,kf.	641,548
11	((“60*” or “61*” or “62*” or “63*” or “64*” or “65*” or “66*” or “67*” or “68*” or “69*”) adj2 (year* or age* or yr*)).tw,kf.	710,721
12	((“thirty-five*” or “thirty-six*” or “thirty-seven*” or “thirty-eight*” or “thirty-nine*”) adj2 (year* or age* or yr*)).tw,kf.	4598
13	((“forty*” or “forty-one*” or “forty-two*” or “forty-three*” or “forty-four*” or “forty-five*” or “forty-six*” or “forty-seven*” or “forty-eight*” or “forty-nine*”) adj2 (year* or age* or yr*)).tw,kf.	9359
14	((“fifty*” or “fifty-one*” or “fifty-two*” or “fifty-three*” or “fifty-four*” or “fifty-five*” or “fifty-six*” or “fifty-seven*” or “fifty-eight*” or “fifty-nine*”) adj2 (year* or age* or yr*)).tw,kf.	10,704
15	((“sixty*” or “sixty-one*” or “sixty-two*” or “sixty-three*” or “sixty-four*” or “sixty-five*” or “sixty-six*” or “sixty-seven*” or “sixty-eight*” or “sixty-nine*”) adj2 (year* or age* or yr*)).tw,kf.	6802
16	or/4–15	6,564,609
17	((masters* or competitive or endurance or elite or ultra*) adj3 (para* or athlete* or running or runner* or cyclist* or cycling or swim* or triathl* or row* or ultra*)).tw,kf.	986,431
18	(endurance adj3 train*).tw,kf.	9597
19	or/17–18	993,294
20	athletic performance/or physical endurance/	35,185
21	Lactic Acid/	48,134
22	oxygen consumption/or anaerobic threshold/	110,805
23	physical fitness/or cardiorespiratory fitness/	33,295
24	Heart Rate/	178,602
25	((aerobic or anaerobic) adj2 (capacity or power or threshold)).tw,kf.	14,822
26	((HR or VO2) adj2 (submax or max or peak)).tw,kf.	8875
27	(critical adj2 (power or speed)).tw,kf.	1642
28	((exercise or running or cycling or swimming) adj2 (economy or efficiency)).tw,kf.	2202
29	(“VO2*” or “oxygen consumption” or “heart rate” or “lactate threshold” or “blood lactate” or “power output” or “energy cost” or “oxygen cost”).tw,kf.	260,771
30	or/20–29	496,507
31	3 and 16 and 19 and 30	4086
32	Ultrasonography/	205,431
33	(ultrasound* or ultrasonograph*).tw,kf.	440,960
34	32 or 33	523,287
35	31 not 34	2677

**Table A2:** Database: Embase 1974 to 2024 September 13. Search Strategy.

#	Searches	Results
1	Female/	12,276,790
2	(female* or women* or woman*).tw,kf.	3,811,953
3	1 or 2	12,698,581
4	Age/or Aging/or Middle Aged/	3,044,145
5	(aging or older).tw,kf.	1,109,141
6	menopause/or climacterium/or postmenopause/	131,335
7	(“transitional menopause” or menopaus* or perimenopaus* or postmenopaus*).tw,kf.	163,631
8	((“35*” or “36*” or “37*” or “38*” or “39*”) adj2 (year* or age* or yr*)).tw,kf.	519,167
9	((“40*” or “41*” or “42*” or “43*” or “44*” or “45*” or “46*” or “47*” or “48*” or “49*”) adj2 (year* or age* or yr*)).tw,kf.	1,068,431
10	((“50*” or “51*” or “52*” or “53*” or “54*” or “55*” or “56*” or “57*” or “58*” or “59*”) adj2 (year* or age* or yr*)).tw,kf.	1,164,724
11	((“60*” or “61*” or “62*” or “63*” or “64*” or “65*” or “66*” or “67*” or “68*” or “69*”) adj2 (year* or age* or yr*)).tw,kf.	1,291,994
12	((“thirty-five*” or “thirty-six*” or “thirty-seven*” or “thirty-eight*” or “thirty-nine*”) adj2 (year* or age* or yr*)).tw,kf.	6531
13	((“forty*” or “forty-one*” or “forty-two*” or “forty-three*” or “forty-four*” or “forty-five*” or “forty-six*” or “forty-seven*” or “forty-eight*” or “forty-nine*”) adj2 (year* or age* or yr*)).tw,kf.	13,271
14	((“fifty*” or “fifty-one*” or “fifty-two*” or “fifty-three*” or “fifty-four*” or “fifty-five*” or “fifty-six*” or “fifty-seven*” or “fifty-eight*” or “fifty-nine*”) adj2 (year* or age* or yr*)).tw,kf.	14,296
15	((“sixty*” or “sixty-one*” or “sixty-two*” or “sixty-three*” or “sixty-four*” or “sixty-five*” or “sixty-six*” or “sixty-seven*” or “sixty-eight*” or “sixty-nine*”) adj2 (year* or age* or yr*)).tw,kf.	9980
16	or/4–15	5,961,766
17	((masters* or competitive or endurance or elite or ultra*) adj3 (para* or athlete* or running or runner* or cyclist* or cycling or swim* or triathl* or row* or ultra*)).tw,kf.	1,263,681
18	(endurance adj3 train*).tw,kf.	12,321
19	endurance athlete/	814
20	or/17–19	1,272,835
21	athletic performance/or endurance/	44,830
22	lactate blood level/or oxygen consumption/or anaerobic threshold/	135,959
23	fitness/or cardiorespiratory fitness/	53,887
24	heart rate/	284,617
25	((aerobic or anaerobic) adj2 (capacity or power or threshold)).tw,kf.	19,582
26	((HR or VO2) adj2 (submax or max or peak)).tw,kf.	16,119
27	(critical adj2 (power or speed)).tw,kf.	1669
28	((exercise or running or cycling or swimming) adj2 (economy or efficiency)).tw,kf.	2362
29	(“VO2*” or “oxygen consumption” or “heart rate” or “lactate threshold” or “blood lactate” or “power output” or “energy cost” or “oxygen cost”).tw,kf.	354,535
30	or/21–29	611,099
31	3 and 16 and 20 and 30	5188
32	Ultrasound/or echography/	635,526
33	(ultrasound* or ultrasonograph*).tw,kf.	678,518
34	32 or 33	933,466
35	31 not 34	2673

**Table A3:** Database: CINAHL 1974 to 2024 September 16. Search Strategy.

#	Query	Results
S1	(MH “Female”)	(2,277,662)
S2	TI (female* or women* or woman*) OR AB (female* or women* or woman*)	(727,716)
S3	S1 OR S2	(2,436,750)
S4	(MH “Age Factors”) or (MH “Aging”) or (MH “Middle Age”)	(1,290,360)
S5	TI (aging or older) OR AB (aging or older)	(298,217)
S6	(MH “Menopause”) OR (MH “Perimenopause”) OR (MH “Postmenopause”)	(23,403)
S7	TI (“transitional menopause” or menopaus* or perimenopaus* or postmenopaus*) OR AB (“transitional menopause” or menopaus* or perimenopaus* or postmenopaus*)	(33,771)
S8	TI ((“35*” or “36*” or “37*” or “38*” or “39*”) N2 (year* or age* or yr*)) OR AB ((“35*” or “36*” or “37*” or “38*” or “39*”) N2 (year* or age* or yr*))	(64,044)
S9	TI ((“40*” or “41*” or “42*” or “43*” or “44*” or “45*” or “46*” or “47*” or “48*” or “49*”) N2 (year* or age* or yr*)) OR AB ((“40*” or “41*” or “42*” or “43*” or “44*” or “45*” or “46*” or “47*” or “48*” or “49*”) N2 (year* or age* or yr*))	(132,260)
S10	TI ((“50*” or “51*” or “52*” or “53*” or “54*” or “55*” or “56*” or “57*” or “58*” or “59*”) N2 (year* or age* or yr*)) OR AB ((“50*” or “51*” or “52*” or “53*” or “54*” or “55*” or “56*” or “57*” or “58*” or “59*”) N2 (year* or age* or yr*))	(144,225)
S11	TI ((“60*” or “61*” or “62*” or “63*” or “64*” or “65*” or “66*” or “67*” or “68*” or “69*”) N2 (year* or age* or yr*)) OR AB ((“60*” or “61*” or “62*” or “63*” or “64*” or “65*” or “66*” or “67*” or “68*” or “69*”) N2 (year* or age* or yr*))	(166,916)
S12	TI ((“thirty-five*” or “thirty-six*” or “thirty-seven*” or “thirty-eight*” or “thirty-nine*”) N2 (year* or age* or yr*)) OR AB ((“thirty-five*” or “thirty-six*” or “thirty-seven*” or “thirty-eight*” or “thirty-nine*”) N2 (year* or age* or yr*))	(1801)
S13	TI ((“forty*” or “forty-one*” or “forty-two*” or “forty-three*” or “forty-four*” or “forty-five*” or “forty-six*” or “forty-seven*” or “forty-eight*” or “forty-nine*”) N2 (year* or age* or yr*)) OR AB ((“forty*” or “forty-one*” or “forty-two*” or “forty-three*” or “forty-four*” or “forty-five*” or “forty-six*” or “forty-seven*” or “forty-eight*” or “forty-nine*”) N2 (year* or age* or yr*))	(4429)
S14	TI ((“fifty*” or “fifty-one*” or “fifty-two*” or “fifty-three*” or “fifty-four*” or “fifty-five*” or “fifty-six*” or “fifty-seven*” or “fifty-eight*” or “fifty-nine*”) N2 (year* or age* or yr*)) OR AB ((“fifty*” or “fifty-one*” or “fifty-two*” or “fifty-three*” or “fifty-four*” or “fifty-five*” or “fifty-six*” or “fifty-seven*” or “fifty-eight*” or “fifty-nine*”) N2 (year* or age* or yr*))	(4216)
S15	TI ((“sixty*” or “sixty-one*” or “sixty-two*” or “sixty-three*” or “sixty-four*” or “sixty-five*” or “sixty-six*” or “sixty-seven*” or “sixty-eight*” or “sixty-nine*”) N2 (year* or age* or yr*)) OR AB ((“sixty*” or “sixty-one*” or “sixty-two*” or “sixty-three*” or “sixty-four*” or “sixty-five*” or “sixty-six*” or “sixty-seven*” or “sixty-eight*” or “sixty-nine*”) N2 (year* or age* or yr*))	(3414)
S16	S4 OR S5 OR S6 OR S7 OR S8 OR S9 OR S10 OR S11 OR S12 OR S13 OR S14 OR S15	(1,618,684)
S17	TI ((masters* or competitive or endurance or elite or ultra*) N3 (para* or athlete* or running or runner* or cyclist* or cycling or swim* or triathl* or row* or ultra*)) OR AB ((masters* or competitive or endurance or elite or ultra*) N3 (para* or athlete* or running or runner* or cyclist* or cycling or swim* or triathl* or row* or ultra*))	(13,914)
S18	TI (endurance N3 train*) OR AB (endurance N3 train*)	(3170)
S19	(MH “Athletes, Masterss”)	(265)
S20	S17 OR S18 OR S19	(16,203)
S21	(MH “Athletic Performance) OR (MH “Endurance Sports”) OR (MH “Physical Endurance”)	(14,088)
S22	(MH “Lactic Acid”)	(3465)
S23	(MH “Oxygen Consumption”) OR (MH “Anaerobic Threshold”)	(19,243)
S24	(MH “Physical Fitness”) OR (MH “Cardiorespiratory Fitness”)	(21,890)
S25	(MH “Heart Rate”)	(34,225)
S26	TI ((aerobic or anaerobic) N2 (capacity or power or threshold)) OR AB ((aerobic or anaerobic) N2 (capacity or power or threshold))	(4456)
S27	TI ((HR or VO2) N2 (submax or max or peak)) OR AB ((HR or VO2) N2 (submax or max or peak))	(2183)
S28	TI (critical N2 (power or speed)) OR AB (critical N2 (power or speed))	(504)
S29	TI ((exercise or running or cycling or swimming) N2 (economy or efficiency)) OR AB ((exercise or running or cycling or swimming) N2 (economy or efficiency))	(834)
S30	TI (“VO2*” or “oxygen consumption” or “heart rate” or “lactate threshold” or “blood lactate” or “power output” or “energy cost” or “oxygen cost”) OR AB (“VO2*” or “oxygen consumption” or “heart rate” or “lactate threshold” or “blood lactate” or “power output” or “energy cost” or “oxygen cost”)	(50,803)
S31	S21 OR S22 OR S23 OR S24 OR S25 OR S26 OR S27 OR S28 OR S29 OR S30	(96,185)
S32	S3 AND S16 AND S20 AND S31	(452)
S33	(MH “Ultrasonography”)	(44,980)
S34	TI (ultrasound* or ultrasonograph*) OR AB (ultrasound* or ultrasonograph*)	(99,021)
S35	S33 OR S34	(115,612)
S36	S32 not S35	(430)

**Table A4:** Database: SPORTdiscus 1974 to 2024 September 16. Search Strategy.

#	Query	Results
S1	DE “WOMEN” OR DE “WOMEN athletes” OR DE “WOMEN track & field athletes” OR DE “WOMEN rowers” OR DE “WOMEN long-distance runners” OR DE “WOMEN cyclists” OR DE “WOMEN endurance athletes”	(53,597)
S2	TI (female* or women* or woman*) OR AB (female* or women* or woman*) OR KW (female* or women* or woman*)	(203,783)
S3	S1 OR S2	(225,862)
S4	DE “AGING”	(10,603)
S5	TI (age factors or aging or older or “middle age*”) OR AB (age factors or aging or older or “middle age*”) OR KW (age factors or aging or older or “middle age*”)	(76,376)
S6	DE “MENOPAUSE” OR DE “PERIMENOPAUSE”	(1668)
S7	TI (“transitional menopause” or menopaus* or perimenopaus* or postmenopaus*) OR AB (“transitional menopause” or menopaus* or perimenopaus* or postmenopaus*) OR KW (“transitional menopause” or menopaus* or perimenopaus* or postmenopaus*)	(4612)
S8	TI ((“35*” or “36*” or “37*” or “38*” or “39*”) N2 (year* or age* or yr*)) OR AB ((“35*” or “36*” or “37*” or “38*” or “39*”) N2 (year* or age* or yr*)) OR KW ((“35*” or “36*” or “37*” or “38*” or “39*”) N2 (year* or age* or yr*))	(10,853)
S9	TI ((“40*” or “41*” or “42*” or “43*” or “44*” or “45*” or “46*” or “47*” or “48*” or “49*”) N2 (year* or age* or yr*)) OR AB ((“40*” or “41*” or “42*” or “43*” or “44*” or “45*” or “46*” or “47*” or “48*” or “49*”) N2 (year* or age* or yr*)) OR KW ((“40*” or “41*” or “42*” or “43*” or “44*” or “45*” or “46*” or “47*” or “48*” or “49*”) N2 (year* or age* or yr*))	(21,225)
S10	TI ((“50*” or “51*” or “52*” or “53*” or “54*” or “55*” or “56*” or “57*” or “58*” or “59*”) N2 (year* or age* or yr*)) OR AB ((“50*” or “51*” or “52*” or “53*” or “54*” or “55*” or “56*” or “57*” or “58*” or “59*”) N2 (year* or age* or yr*)) OR KW ((“50*” or “51*” or “52*” or “53*” or “54*” or “55*” or “56*” or “57*” or “58*” or “59*”) N2 (year* or age* or yr*))	(21,014)
S11	TI ((“60*” or “61*” or “62*” or “63*” or “64*” or “65*” or “66*” or “67*” or “68*” or “69*”) N2 (year* or age* or yr*)) OR AB ((“60*” or “61*” or “62*” or “63*” or “64*” or “65*” or “66*” or “67*” or “68*” or “69*”) N2 (year* or age* or yr*)) OR KW ((“60*” or “61*” or “62*” or “63*” or “64*” or “65*” or “66*” or “67*” or “68*” or “69*”) N2 (year* or age* or yr*))	(22,212)
S12	TI ((“thirty-five*” or “thirty-six*” or “thirty-seven*” or “thirty-eight*” or “thirty-nine*”) N2 (year* or age* or yr*)) OR AB ((“thirty-five*” or “thirty-six*” or “thirty-seven*” or “thirty-eight*” or “thirty-nine*”) N2 (year* or age* or yr*)) OR KW ((“thirty-five*” or “thirty-six*” or “thirty-seven*” or “thirty-eight*” or “thirty-nine*”) N2 (year* or age* or yr*))	(589)
S13	TI ((“forty*” or “forty-one*” or “forty-two*” or “forty-three*” or “forty-four*” or “forty-five*” or “forty-six*” or “forty-seven*” or “forty-eight*” or “forty-nine*”) N2 (year* or age* or yr*)) OR AB ((“forty*” or “forty-one*” or “forty-two*” or “forty-three*” or “forty-four*” or “forty-five*” or “forty-six*” or “forty-seven*” or “forty-eight*” or “forty-nine*”) N2 (year* or age* or yr*)) OR KW ((“forty*” or “forty-one*” or “forty-two*” or “forty-three*” or “forty-four*” or “forty-five*” or …	(1393)
S14	TI ((“fifty*” or “fifty-one*” or “fifty-two*” or “fifty-three*” or “fifty-four*” or “fifty-five*” or “fifty-six*” or “fifty-seven*” or “fifty-eight*” or “fifty-nine*”) N2 (year* or age* or yr*)) OR AB ((“fifty*” or “fifty-one*” or “fifty-two*” or “fifty-three*” or “fifty-four*” or “fifty-five*” or “fifty-six*” or “fifty-seven*” or “fifty-eight*” or “fifty-nine*”) N2 (year* or age* or yr*)) OR KW ((“fifty*” or “fifty-one*” or “fifty-two*” or “fifty-three*” or “fifty-four*” or “fifty-five*” or …	(1525)
S15	TI ((“sixty*” or “sixty-one*” or “sixty-two*” or “sixty-three*” or “sixty-four*” or “sixty-five*” or “sixty-six*” or “sixty-seven*” or “sixty-eight*” or “sixty-nine*”) N2 (year* or age* or yr*)) OR AB ((“sixty*” or “sixty-one*” or “sixty-two*” or “sixty-three*” or “sixty-four*” or “sixty-five*” or “sixty-six*” or “sixty-seven*” or “sixty-eight*” or “sixty-nine*”) N2 (year* or age* or yr*)) OR KW ((“sixty*” or “sixty-one*” or “sixty-two*” or “sixty-three*” or “sixty-four*” or “sixty-five*” or …	(1112)
S16	S4 OR S5 OR S6 OR S7 OR S8 OR S9 OR S10 OR S11 OR S12 OR S13 OR S14 OR S15	(126,004)
S17	(DE “WOMEN’S sports” OR DE “WOMEN’S cycling” OR DE “WOMEN’S rowing” OR DE “WOMEN’S running” OR DE “WOMEN’S track & field”) OR (DE “TRIATHLONS for women”) OR (DE “MARATHON running for women”)	(3253)
S18	TI ((masters* or competitive or endurance or elite or ultra*) N3 (para* or athlete* or running or runner* or cyclist* or cycling or swim* or triathl* or row* or ultra*)) OR AB ((masters* or competitive or endurance or elite or ultra*) N3 (para* or athlete* or running or runner* or cyclist* or cycling or swim* or triathl* or row* or ultra*)) OR KW ((masters* or competitive or endurance or elite or ultra*) N3 (para* or athlete* or running or runner* or cyclist* or cycling or swim* or triathl* or …	(47,452)
S19	TI endurance N3 train* OR AB endurance N3 train* OR KW endurance N3 train*	(7713)
S20	S17 OR S18 OR S19	(55,792)
S21	DE “WOMEN athletes’ physiology”	(126)
S22	DE “ANAEROBIC threshold” OR DE “BLOOD lactate” OR DE “LACTIC acid”	(5154)
S23	DE “OXYGEN consumption” OR DE “AEROBIC capacity”	(17,009)
S24	DE “PHYSICAL fitness” OR DE “CARDIOVASCULAR fitness” OR DE “CARDIOPULMONARY fitness”	(92,858)
S25	DE “HEART beat”	(19,415)
S26	TI ((aerobic or anaerobic) N2 (capacity or power or threshold)) OR AB ((aerobic or anaerobic) N2 (capacity or power or threshold)) OR KW ((aerobic or anaerobic) N2 (capacity or power or threshold))	(17,044)
S27	TI ((HR or VO2) N2 (submax or max or peak)) OR AB ((HR or VO2) N2 (submax or max or peak)) OR KW ((HR or VO2) N2 (submax or max or peak))	(4396)
S28	TI ((critical N2 (power or speed)) OR AB ((critical N2 (power or speed)) OR KW ((critical N2 (power or speed))	(803)
S29	TI ((exercise or running or cycling or swimming) N2 (economy or efficiency)) OR AB ((exercise or running or cycling or swimming) N2 (economy or efficiency)) OR KW ((exercise or running or cycling or swimming) N2 (economy or efficiency))	(2099)
S30	TI (“VO2*” or “oxygen consumption” or “heart rate” or “lactate threshold” or “blood lactate” or “power output” or “energy cost” or “oxygen cost”) OR AB (“VO2*” or “oxygen consumption” or “heart rate” or “lactate threshold” or “blood lactate” or “power output” or “energy cost” or “oxygen cost”) OR KW (“VO2*” or “oxygen consumption” or “heart rate” or “lactate threshold” or “blood lactate” or “power output” or “energy cost” or “oxygen cost”)	(45,989)
S31	S21 OR S22 OR S23 OR S24 OR S25 OR S26 OR S27 OR S28 OR S29 OR S30	(147,529)
S32	S3 AND S16 AND S20 AND S31	(427)
S33	DE “ULTRASONIC imaging” OR DE “ULTRASONICS”	(3362)
S34	TI (ultrasound* or ultrasonograph*) OR AB (ultrasound* or ultrasonograph*) OR KW (ultrasound* or ultrasonograph*)	(9936)
S35	S33 OR S34	(10,595)
S36	S32 not S35	(418)

**Table A5:** Database: Scopus 1974 to 2024 September 16. Search Strategy.

String	Results
(((TITLE-ABS-KEY (((aerobic OR anaerobic) W/2 (capacity OR power OR threshold))) OR (TITLE-ABS-KEY ((hr OR vo2) W/2 (submax OR max OR peak)))) OR (TITLE-ABS-KEY ((critical W/2 (power OR speed)))) OR (TITLE-ABS-KEY (((exercise OR running OR cycling OR swimming) W/2 (economy OR efficiency)))) OR (TITLE-ABS-KEY ((“VO2*” OR “oxygen consumption” OR “heart rate” OR “lactate threshold” OR “blood lactate” OR “power output” OR “energy cost” OR “oxygen cost”))))) AND (TITLE-ABS-KEY (((masters* OR competitive OR endurance OR elite OR ultra*) W/3 (para* OR athlete* OR running OR runner* OR cyclist* OR cycling OR swim* OR triathl* OR row* OR ultra*))) OR (TITLE-ABS-KEY ((endurance W/3 train*)))) AND (((TITLE-ABS-KEY ((aging OR older)) OR (TITLE-ABS-KEY ((“transitional menopause” OR menopaus* OR perimenopaus* OR postmenopaus*))) OR (TITLE-ABS-KEY (((“35*” OR “36*” OR “37*” OR “38*” OR “39*”) W/2 (year* OR age* OR yr*)))) OR (TITLE-ABS-KEY (((“40*” OR “41*” OR “42*” OR “43*” OR “44*” OR “45*” OR “46*” OR “47*” OR “48*” OR “49*”) W/2 (year* OR age* OR yr*)))) OR (TITLE-ABS-KEY (((“50*” OR “51*” OR “52*” OR “53*” OR “54*” OR “55*” OR “56*” OR “57*” OR “58*” OR “59*”) W/2 (year* OR age* OR yr*)))) OR (TITLE-ABS-KEY (((“60*” OR “61*” OR “62*” OR “63*” OR “64*” OR “65*” OR “66*” OR “67*” OR “68*” OR “69*”) W/2 (year* OR age* OR yr*)))))) OR ((TITLE-ABS-KEY (((“thirty-five*” OR “thirty-six*” OR “thirty-seven*” OR “thirty-eight*” OR “thirty-nine*”) W/2 (year* OR age* OR yr*))) OR (TITLE-ABS-KEY (((“forty*” OR “forty-one*” OR “forty-two*” OR “forty-three*” OR “forty-four*” OR “forty-five*” OR “forty-six*” OR “forty-seven*” OR “forty-eight*” OR “forty-nine*”) W/2 (year* OR age* OR yr*)))) OR (TITLE-ABS-KEY (((“fifty*” OR “fifty-one*” OR “fifty-two*” OR “fifty-three*” OR “fifty-four*” OR “fifty-five*” OR “fifty-six*” OR “fifty-seven*” OR “fifty-eight*” OR “fifty-nine*”) W/2 (year* OR age* OR yr*)))) OR (TITLE-ABS-KEY (((“sixty*” OR “sixty-one*” OR “sixty-two*” OR “sixty- three*” OR “sixty-four*” OR “sixty-five*” OR “sixty-six*” OR “sixty-seven*” OR “sixty-eight*” OR “sixty-nine*”) W/2 (year* OR age* OR yr*))))))) AND (TITLE-ABS-KEY ((female* OR women* OR woman*))) AND NOT (TITLE-ABS-KEY ((ultrasound* OR ultrasonograph*)))	1594

**Table A6:** Database: CENTRAL 1974 to 2024 September 19. Search Strategy.

ID	Search	Hits
#1	([mh ^Female]):ti,ab,kw (Word variations have been searched) in Trials	610,339
#2	(female*:ti,ab,kw OR women*:ti,ab,kw OR woman*:ti,ab,kw) in Trials	1,047,798
#3	#1 OR #2 in Trials	1,047,798
#4	[mh ^”Age Factors”] OR [mh ^Aging] OR [mh ^”Middle Aged”] in Trials	418,369
#5	(aging:ti,ab,kw OR older:ti,ab,kw) in Trials	89,963
#6	[mh ^menopause] OR [mh ^perimenopause] OR [mh ^postmenopause] in Trials	8271
#7	(“transitional menopause”:ti,ab,kw OR menopaus*:ti,ab,kw OR perimenopaus*:ti,ab,kw OR postmenopaus*:ti,ab,kw) in Trials	32,414
#8	((35*:ti,ab,kw OR 36*:ti,ab,kw OR 37*:ti,ab,kw OR 38*:ti,ab,kw OR 39*:ti,ab,kw) NEAR/2 (year*:ti,ab,kw OR age*:ti,ab,kw OR yr*:ti,ab,kw)) in Trials	42,169
#9	((40*:ti,ab,kw OR 41*:ti,ab,kw OR 42*:ti,ab,kw OR 43*:ti,ab,kw OR 44*:ti,ab,kw OR 45*:ti,ab,kw OR 46*:ti,ab,kw OR 47*:ti,ab,kw OR 48*:ti,ab,kw OR 49*:ti,ab,kw) NEAR/2 (year*:ti,ab,kw OR age*:ti,ab,kw OR yr*:ti,ab,kw)) in Trials	91,223
#10	((50*:ti,ab,kw OR 51*:ti,ab,kw OR 52*:ti,ab,kw OR 53*:ti,ab,kw OR 54*:ti,ab,kw OR 55*:ti,ab,kw OR 56*:ti,ab,kw OR 57*:ti,ab,kw OR 58*:ti,ab,kw OR 59*:ti,ab,kw) NEAR/2 (year*:ti,ab,kw OR age*:ti,ab,kw OR yr*:ti,ab,kw)) in Trials	95,565
#11	((60*:ti,ab,kw OR 61*:ti,ab,kw OR 62*:ti,ab,kw OR 63*:ti,ab,kw OR 64*:ti,ab,kw OR 65*:ti,ab,kw OR 66*:ti,ab,kw OR 67*:ti,ab,kw OR 68*:ti,ab,kw OR 69*:ti,ab,kw) NEAR/2 (year*:ti,ab,kw OR age*:ti,ab,kw OR yr*:ti,ab,kw)) in Trials	154,351
#12	((thirty-five*:ti,ab,kw OR thirty-six*:ti,ab,kw OR thirty-seven*:ti,ab,kw OR thirty-eight*:ti,ab,kw OR thirty-nine*:ti,ab,kw) NEAR/2 (year*:ti,ab,kw OR age*:ti,ab,kw OR yr*:ti,ab,kw)) in Trials	580
#13	((forty*:ti,ab,kw OR forty-one*:ti,ab,kw OR forty-two*:ti,ab,kw OR forty-three*:ti,ab,kw OR forty-four*:ti,ab,kw OR forty-five*:ti,ab,kw OR forty-six*:ti,ab,kw OR forty-seven*:ti,ab,kw OR forty-eight*:ti,ab,kw OR forty-nine*:ti,ab,kw) NEAR/2 (year*:ti,ab,kw OR age*:ti,ab,kw OR yr*:ti,ab,kw)) in Trials	1686
#14	((fifty*:ti,ab,kw OR fifty-one*:ti,ab,kw OR fifty-two*:ti,ab,kw OR fifty-three*:ti,ab,kw OR fifty-four*:ti,ab,kw OR fifty-five*:ti,ab,kw OR fifty-six*:ti,ab,kw OR fifty-seven*:ti,ab,kw OR fifty-eight*:ti,ab,kw OR fifty-nine*:ti,ab,kw) NEAR/2 (year*:ti,ab,kw OR age*:ti,ab,kw OR yr*:ti,ab,kw)) in Trials	1291
#15	((sixty*:ti,ab,kw OR sixty-one*:ti,ab,kw OR sixty-two*:ti,ab,kw OR sixty-three*:ti,ab,kw OR sixty-four*:ti,ab,kw OR sixty-five*:ti,ab,kw OR sixty-six*:ti,ab,kw OR sixty-seven*:ti,ab,kw OR sixty-eight*:ti,ab,kw OR sixty-nine*:ti,ab,kw) NEAR/2 (year*:ti,ab,kw OR age*:ti,ab,kw OR yr*:ti,ab,kw)) in Trials	1559
#16	#4 OR #5 OR #6 OR #7 OR #8 OR #9 OR #10 OR #11 OR #12 OR #13 OR #14 OR #15 in Trials	717,868
#17	((masters*:ti,ab,kw OR competitive:ti,ab,kw OR endurance:ti,ab,kw OR elite:ti,ab,kw OR ultra*:ti,ab,kw) NEAR/3 (para*:ti,ab,kw OR athlete*:ti,ab,kw OR running:ti,ab,kw OR runner*:ti,ab,kw OR cyclist*:ti,ab,kw OR cycling:ti,ab,kw OR swim*:ti,ab,kw OR triathl*:ti,ab,kw OR row*:ti,ab,kw OR ultra*:ti,ab,kw)) in Trials	79,971
#18	(endurance:ti,ab,kw NEAR/3 train*:ti,ab,kw) in Trials	4217
#19	#17 OR #18 in Trials	83,284
#20	[mh ^“athletic performance”] OR [mh ^”physical endurance”] in Trials	6341
#21	[mh ^“Lactic Acid”] in Trials	2957
#22	[mh ^“oxygen consumption”] OR [mh ^”anaerobic threshold”] in Trials	8390
#23	[mh ^“physical fitness”] OR [mh ^”cardiorespiratory fitness”] in Trials	4374
#24	[mh ^“Heart Rate”] in Trials	23,780
#25	((aerobic:ti,ab,kw OR anaerobic:ti,ab,kw) NEAR/2 (capacity:ti,ab,kw OR power:ti,ab,kw OR threshold:ti,ab,kw)) in Trials	6081
#26	((HR:ti,ab,kw OR VO2:ti,ab,kw) NEAR/2 (submax:ti,ab,kw OR max:ti,ab,kw OR peak:ti,ab,kw)) in Trials	3657
#27	(critical:ti,ab,kw NEAR/2 (power:ti,ab,kw OR speed:ti,ab,kw)) in Trials	135
#28	((exercise:ti,ab,kw OR running:ti,ab,kw OR cycling:ti,ab,kw OR swimming:ti,ab,kw) NEAR/2 (economy:ti,ab,kw OR efficiency:ti,ab,kw)) in Trials	503
#29	(VO2*:ti,ab,kw OR “oxygen consumption”:ti,ab,kw OR “heart rate”:ti,ab,kw OR “lactate threshold”:ti,ab,kw OR “blood lactate”:ti,ab,kw OR “power output”:ti,ab,kw OR “energy cost”:ti,ab,kw OR “oxygen cost”:ti,ab,kw) in Trials	92,125
#30	#20 OR #21 OR #22 OR #23 OR #24 OR #25 OR #26 OR #27 OR #28 OR #29 in Trials	100,544
#31	#3 AND #16 AND #19 AND #30 in Trials	1629
#32	[mh ^Ultrasonography] in Trials	6544
#33	(ultrasound*:ti,ab,kw OR ultrasonograph*:ti,ab,kw) in Trials	58,392
#34	#32 OR #33 in Trials	58,392
#35	#31 NOT #34 in Trials	883

## Appendix C. Data Extraction Template

Title			Author		Year	Country		Study Design			Sampling Method and Size				Sample Size	
Study #1																
Study #2																
Author	Age		Menopause Status (and Method of Determining Menopause Status)			Menopause Hormone Therapy Users (Y/N)			Sport(s) and Training Status		Competitive Tier [26].	Concept	Context	Primary objective		Secondary objective(s)
									
									
Author	Exercise Protocol		Aerobic Capacity (Y/N)	Aerobic Capacity Equipment and Protocol		Aerobic Capacity Results	Other Performance Metric and Methods #1			Results for Other Performance Metric #1	Other Performance Metric and Methods #2	Results for Other Performance Metric #2		Other Performance Metric and Methods #3		Results for Other Performance Metric #3
										
										
Author		Key Finding(s)			Key Point(s) from Discussion					Conclusion	Limitations		Directions for Future Research			
					
					
																
